# Global, Regional, and National Prevalence of Gout From 1990 to 2019: Age-Period-Cohort Analysis With Future Burden Prediction

**DOI:** 10.2196/45943

**Published:** 2023-06-07

**Authors:** Qiyu He, Tsz-Ngai Mok, Tat-Hang Sin, Jiaying Yin, Sicun Li, Yiyue Yin, Wai-Kit Ming, Bin Feng

**Affiliations:** 1 State Key Laboratory of Cardiovascular Disease, Fuwai Hospital National Center for Cardiovascular Diseases Chinese Academy of Medical Sciences, Peking Union Medical College Bejing China; 2 Department of Infectious Diseases and Public Health Jockey Club College of Veterinary Medicine and Life Sciences City University of Hong Kong Hong Kong Hong Kong; 3 Department of Orthopedic Surgery and Sports Medicine Center The First Affiliated Hospital and The First Clinical College Jinan University Guangzhou China; 4 Department of Surgery Peking Union Medical College Hospital Chinese Academy of Medical Sciences and Peking Union Medical College Beijing China; 5 School of Public Health University of Hong Kong Hong Kong Hong Kong; 6 School of Humanities and Communication Guangdong University of Finance and Economics Guangzhou China; 7 Department of Biostatistics College of Science City University of Hong Kong Hong Kong Hong Kong; 8 School of Public Policy and Management Tsinghua University Beijing China; 9 Department of Orthopedic Surgery Peking Union Medical College Hospital Chinese Academy of Medical Sciences and Peking Union Medical College Beijing China

**Keywords:** gout, prevalence, age-period-cohort analysis, Global Burden of Disease Study 2019, prediction, Bayesian age-period-cohort analysis, Norped age-period-cohort analysis

## Abstract

**Background:**

Gout is a common and debilitating condition that is associated with significant morbidity and mortality. Despite advances in medical treatment, the global burden of gout continues to increase, particularly in high–sociodemographic index (SDI) regions.

**Objective:**

To address the aforementioned issue, we used age-period-cohort (APC) modeling to analyze global trends in gout incidence and prevalence from 1990 to 2019.

**Methods:**

Data were extracted from the Global Burden of Disease Study 2019 to assess all-age prevalence and age-standardized prevalence rates, as well as years lived with disability rates, for 204 countries and territories. APC effects were also examined in relation to gout prevalence. Future burden prediction was carried out using the *Nordpred* APC prediction of future incidence cases and the Bayesian APC model.

**Results:**

The global gout incidence has increased by 63.44% over the past 2 decades, with a corresponding increase of 51.12% in global years lived with disability. The sex ratio remained consistent at 3:1 (male to female), but the global gout incidence increased in both sexes over time. Notably, the prevalence and incidence of gout were the highest in high-SDI regions (95% uncertainty interval 14.19-20.62), with a growth rate of 94.3%. Gout prevalence increases steadily with age, and the prevalence increases rapidly in high-SDI quantiles for the period effect. Finally, the cohort effect showed that gout prevalence increases steadily, with the risk of morbidity increasing in younger birth cohorts. The prediction model suggests that the gout incidence rate will continue to increase globally.

**Conclusions:**

Our study provides important insights into the global burden of gout and highlights the need for effective management and prophylaxis of this condition. The APC model used in our analysis provides a novel approach to understanding the complex trends in gout prevalence and incidence, and our findings can inform the development of targeted interventions to address this growing health issue.

## Introduction

### Background

Gout is a ubiquitous chronic disease caused by an elevation of urate concentrations in articular and nonarticular structures. It can lead to highly intensive pain, lingering discomfort, and inflammation, as well as limited range of motion at the joints, with a high population burden [1]. With a high recurrence rate, many patients cannot undergo standard therapy, which leads to poor health-related quality of life [2]. Besides the increased personal financial burden, direct and indirect annual financial burdens exist for those who cannot control gout well [3]. Patients with cardiovascular disease who have also been diagnosed with gout have higher mortality rates, regardless of age, sex, and comorbidities factors [4]. The mortality gap of gout, which is the association with premature death, has stayed consistent over 2 decades [5,6].

Despite the heavy population burden, especially in the high–sociodemographic index (SDI) countries, the latest studies only focused on the regional or short-period gout burden [7-10]. Because of technological advancements, people’s lifestyles have changed dramatically over the past 2 decades. The tendency and pattern of gout have changed owing to the lifestyle changes worldwide, even with treatment recommendations by many rheumatological societies. However, health policies have not provided any effective action strategies to cope with the steadily increasing trend of gout incidence [11-13]. To break through the barrier, it is essential to provide different approaches regarding the epidemiology of gout to evaluate public health development over time. In this regard, an analysis of prevalence trends, with a particular focus on their associations with age-period-cohort (APC) effects, has the potential to delineate the success of different aspects of health care delivery and identify the remaining treatment gaps [14,15].

Using the APC model, we can potentially mine ethnic disparities and geographic differences, as well as the effectiveness of the monitoring system. As the comparison of collinearity factors should be determined through the additional constraints [16]. This is a valid model for providing valuable insight into various public health issues. It could compare the efficiency of health care provision of different regions and identify the emblematic countries or regions that have acted efficiently on gout prevalence [17-20].

### Objectives

This study aims to use the APC model to analyze the global-, regional-, and national-level prevalence of gout. The *Nordpred* and Bayesian APC (BAPC) prediction models were used to estimate future gout incidence. Although Jeong et al [21] have analyzed the global burden of gout and its associations with various factors such as sex, age, and region, as well as risk factors such as obesity and potential genetic factors, using Global Burden of Disease (GBD) Study 2019 data, this study aims to analyze global trends in gout incidence and prevalence and discuss local health care delivery to learn what may lead to novel action for better management in the future [21]. Therefore, by analyzing the GBD Study 2019 data from 1990 to 2019 by age, sex, and SDI, we have provided an in-depth gout report.

## Methods

### Overview

All data were extracted from the GBD Study 2019 [22], which provides descriptive epidemiological data on 369 diseases and injuries in 204 countries and territories from 1990 to 2019, including incidence, prevalence, mortality, years of life lost, years lived with disability (YLD), and disability-adjusted life years [23]. To identify the data source from a systematic review of published studies, searches of government and international organization websites, published reports, and primary data sources were undertaken, and librarians reviewed each data source. Diagnosis codes from both the International Classification of Diseases, Ninth Revision (ICD-9; 274.x), and the International Classification of Diseases, Tenth Revision (ICD-10; M10.x), were used to identify cases of gout in the GBD Study 2019 data set. The search terms used included *acute gout*, *gouty arthropathy*, *gouty neuritis*, and *primary gout*. According to the American College of Rheumatology diagnostic criteria for gout, the gold standard consists of checking for the presence of monosodium urate crystals in the synovial fluid of the affected joint.

### Ethical Considerations

This study used data from the GBD Study 2019, which was approved by the institutional review board of the University of Washington School of Medicine. As this is a secondary analysis of existing data, no additional human participant research ethics review or informed consent was required. The original data collection obtained informed consent from study participants or was granted exemptions by the institutional review board. Study data were anonymized and deidentified to protect the privacy and confidentiality of study participants.

### Analysis of Overall Temporal Trends

Using all-age prevalence (number) and age-standardized prevalence rates, we assessed the temporal trend in gout prevalence over the study period from 1990 to 2019. Age-standardized population prevalence was calculated using the global data from the GBD Study 2019. Measuring age-standardized population prevalence can provide a method that compares prevalence among countries and SDI quantiles with different populations. As they provide an indirect insight into epidemiological severity, YLD data were examined using an age-standardized YLD rate for the study period from 1990 to 2019. YLD refers to the number of years that an individual with an illness that affects their quality of life, in this case, a patient with a confirmed diagnosis of gout, survives until death [24].

### APC Analysis

The APC model was used mainly to analyze gout morbidity trends and predict future gout burden. The model considers 3 factors: age, period, and cohort. Time trends of diseases are usually explained by age effects, period effects, and cohort effects, as well as random variation. The critical problem of descriptive epidemiology in this case was to describe the APC results of disease occurrence under the condition of gout rates. In the APC model, the period effect refers to the change of human factors affecting the gout rate in the population, such as the development of disease diagnosis technology, screening and early detection, changes in disease definition and registration, and treatment improvement. These human factors may affect disease rates in different periods, resulting in period effects. The age effect is one of the most critical determinants of disease occurrence. The cohort effect refers to changes in disease rates caused by different levels of exposure to risk factors in different generations. The APC model estimates the APC effects by SDI quintiles. The age effect uses longitudinal and cross-sectional age curves, representing age-related gout prevalence; fitted temporal trends display the period effect, period risk ratio, and period deviations; and cohort effects are displayed using cohort RR, local drifts, and cohort deviations. In the APC model, net drifts represent the overall trend in prevalence or incidence over time, whereas local drifts reflect the trends within a specific time period or age group. Age effects measure the changes in prevalence or incidence associated with aging, period effects refer to changes that affect all age groups at a particular time point, and cohort effects reflect the changes in prevalence or incidence for individuals born in the same year. The model also included the issue of covariance among age, period, and cohort, which was addressed by the identifiability constraint that the sum of the APC coefficients must equal 0. However, this constraint can lead to difficulties in estimating the separate APC effects. To address this issue, the estimable functions approach is used, which involves combining linear constraints on the APC coefficients to estimate these effects. Specifically, the sum-to-0 constraint is used to estimate the net drifts (long-term trends) and local drifts (deviations from the long-term trends) for each effect. The net drifts correspond to the overall change in the outcome over time, whereas the local drifts correspond to the deviations from the overall change. The APC model can thus provide insights into the unique APC effects on gout prevalence and incidence over time. In the case of gout, the model grouped age effect into 10-year intervals, period effect into 5-year intervals, and cohort effect into 5-year intervals to simplify the analysis. The x intercept for the cohort effect is set at the year 1945, and the reference choice (the baseline for comparison) is indicated with a dashed line.

### Prediction Model

To reflect the trends in gout burden, the number of new cases of gout from 2019 to 2042 was predicted using a *Nordpred* APC analysis by sex. The R software (R Foundation for Statistical Computing) package *Nordpred* was used for this analysis, which takes into account changing rates and changing population structures, as demonstrated in previous studies [25]. In addition, to facilitate comparison with the predicted results, we calculated the absolute number of events that would occur if the rates remained stable (baseline reference), decreased by 1% per year (optimistic reference), or increased by 1% per year (pessimistic reference) based on actual observed rates in 2019. To validate the stability of the prediction results, we performed a sensitivity analysis using the BAPC model integrated nested Laplace approximations (INLA) with the *BAPC* and *INLA* packages in R.

### Statistical Analysis

Our study used the APC model to analyze gout morbidity and mortality trends and predict future gout burden. The APC model estimates the time trend of prevalence within each age group, expressed as the annual percentage change of age-specific prevalence, reflecting the birth cohort effect trend, whereas Jeong et al [21] used counts, age-standardized rates, age-standardized percentage changes of prevalence, incidence, and YLD to quantify global trends in the burden of gout using the GBD Study 2019 standard population from 1990 to 2019. The Wald chi-square test results indicate whether there is a birth cohort effect and whether the test results are statistically different. An important implication is that a single age-standardized rate curve and estimated annual percentage change value do not adequately describe the time trend in each age group. A drift absolute value of >1% is considered a material change, which is tested by the Wald chi-square test. All analyses were 2-sided, and statistical significance was considered at *P*<.05. Statistical analysis was conducted using R software (version 3.6.3), and the result was presented using 95% uncertainty intervals (UIs).

## Results

### Global and Regional Trends in Gout Incidence From 1990 to 2019

**Figure 1:**
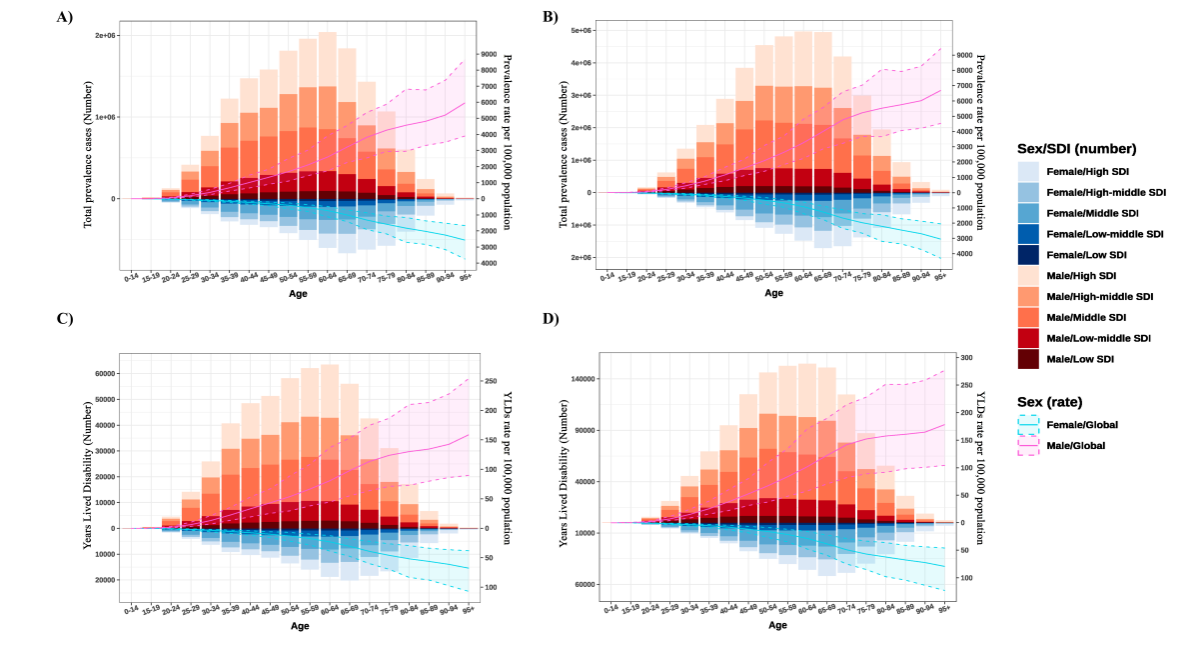
The age effect for gout is shown with all sociodemographic index (SDI) quantiles on total prevalence cases (number) and prevalence rate per 100,000 population from (A) 1990 to (B) 2019. The age effect is shown with all SDI quantiles on years lived with disability (YLD; number) and the YLD rate per 100,000 population from (C) 1990 to (D) 2019. The global tendency under the age effect on both gender.

The global population of individuals with gout increased in the past 30 years from 22 million (95% UI 17.52-27.37) to 53 million (95% UI 43.38-66.34). The growth rate of gout *incidence* was 63.44% (95% UI 57.33%-68.78%). YLD increased from 0.69 million (95% UI 0.44-99.37) to 1.67 million (95% UI 1.07-2.39). The growth rate of global YLD was 51.12% (95% UI 45.91%-56.51%). The sex ratio stayed consistent at 3:1 (male to female), but the gout *incidence* kept increasing over time. The number of male individuals with gout increased from 16 million (95% UI 13.22-20.68) to 40 million (95% UI 32.75-50.10). The growth rate of gout incidence in male individuals was 70.15% (95% UI 64.55%-75.74%). The number of female individuals with gout increased from 5.3 million (95% UI 4.25-6.75) to 13.2 million (95% UI 106.03-163.79), and the growth rate of gout incidence in female individuals was 68.70% (95% UI 63.70%-73.43%). In the high- and middle-SDI regions, the number of individuals with gout skyrocketed from 7 million (95% UI 5.67-8.84) to 17 million (95% UI 14.19-20.62) and from 6 million (95% UI 4.64-7.26) to 15 million (95% UI 12.28-19.40), respectively, with growth rates of 94.3% (95% UI 84.33%-107.15%) and 90.46% (81.34%-98.45%), respectively (Figure 1; Table 1).

**Table 1:** Trends in gout prevalence and years lived with disability (YLD) across sociodemographic index (SDI) quintiles from 1990 to 2019.^a^

			Global, 1990-2019		High SDI, 1990-2019		High-middle SDI, 1990-2019		Middle SDI, 1990-2019		Low-middle SDI, 1990-2019		Low SDI, 1990-2019
**All-age incidence**													
	Cell number×10,000, n (95% UI^b^)	2206.37 (1751.87-2737.37)-5387.18 (4338.32-6634.23)		711.33 (566.90-884.13)-1713.24 (1419.06-2061.89)		538.4 (428.55-671.04)-1239.95 (981.62-1549.99)		585.1 (464.74-726.11)-1549.82 (1228.03-1940.43)		265.61 (210.77-331.26)-635.63 (505.79-796.65)		105.05 (83.22-130.97)-246.51 (196.23-307.16)	
	Annual change, % (95% UI)	63.44 (57.33-68.78)		71.46 (62.62-81.17)		82.60 (75.02-89.65)		74.35 (65.47-82.43)		60.86 (55.38-65.70)		16.23 (13.61-18.94)	
**All-age prevalence rate**													
	Rate per 100,000, n (95% UI)	412.42 (327.46-511.67)-696.25 (857.42-6634.23)		865.35 (689.65-1075.57)-1690.61 (1400.31-2034.66)		468.00 (372.51-583.30)-866.85 (686.26-1083.60)		340.82 (270.71-422.96)-646.68 (512.41-809.67)		235.13 (186.58-293.25)-360.34 (286.73-451.62)		198.91 (157.58-247.98)-246.51 (196.23-307.16)	
	Annual change, % (95% UI)	69.24 (63.79-74.51)		94.32 (84.33-107.15)		84.66 (78.25-89.98)		90.46 (81.34-98.45)		53.25 (50.48-58.90)		11.47 (9.38-13.71)	
**YLD**													
	Cell number×10,000, n (95% UI)	69.12 (43.74-99.37)-167.4 (106.80-239.34)		21.92 (13.93-31.51)-52.03 (33.75-73.50)		16.93 (10.60-24.45)-38.83 (24.16-55.90)		18.61 (11.70-26.86)-48.85 (30.57-70.67)		8.34 (5.24-12.01)-19.88 (12.40-28.57)		3.29 (2.07-4.73)-7.75 (4.84-11.22)	
	Annual change, % (95% UI)	51.12 (45.91-56.51)		65.90 (56.91-77.24)		60.64 (53.55-66.88)		57.33 (48.88-65.49)		43.12 (37.42-49.04)		17.96 (13.66-22.58)	
**APC^c^ model estimates**													
	Annual net drift^d^ of prevalence, % (95% CI)	0.85 (0.80-0.89)		1.596 (1.5332-1.6588)		0.9507 (0.9014-1)		0.7075 (0.6497-0.7654)		0.3088 (0.2879-0.3297)		0.1497 (0.1382-0.1613)	
**Age-standardized prevalence rate**													
	Rate per 100,000, n (95% UI)	532.99 (425.35-657.86)-652.24 (528.56-798.60)		711.83 (565.01-887.95)-1041.01 (862.79-1244.91)		492.64 (393.93-611.45)-624.81 (495.85-778.15)		507.06 (405.34-632.91)-597.19 (476.09-740.10)		403.01 (324.18-502.21)-439.20 (351.05-550.29)		406.42 (324.02-512.66)-424.68 (339.15-532.13)	
	Annual change, % (95% UI)	23.69 (21.65-26.25)		47.95 (40.56-57.67)		28.97 (26.99-30.76)		19.79 (17.53-21.97)		10.56 (9.15-12.03)		5.74 (4.43-7.15)	
**Age-standardized YLD rate**													
	Rate per 100,000, n (95% UI)	16.55 (10.44-23.79)-20.23 (12.91-28.88)		22.03 (13.87-31.73)-32.05 (20.59-44.73)		15.41 (9.67-22.17)-19.62 (12.31-28.20)		15.89 (9.95-22.93)-18.70 (11.89-26.99)		12.44 (7.88-18.03)-13.59 (8.61-19.60)		12.48 (7.93-18.03)-13.11 (8.27-19.07)	
	Annual change, % (95% UI)	25.94 (23.40-28.94)		43.04 (35.31-52.93)		33.70 (29.86-37.29)		20.72 (17.16-24.47)		15.99 (12.58-19.37)		12.68 (9.27-16.21)	

^a^Age-standardized mortality rate is computed by direct standardization with the global standard population in the Global Burden of Disease Study 2019.

^b^UI: uncertainty interval.

^c^APC: age-period-cohort.

^d^Net drifts are estimates derived from the age-period-cohort model and denote overall annual percentage change in mortality, which captures the contribution of the effects from calendar time and successive birth cohorts.

### Global and Regional Trends in Gout Prevalence From 1990 to 2019

**Figure 2:**
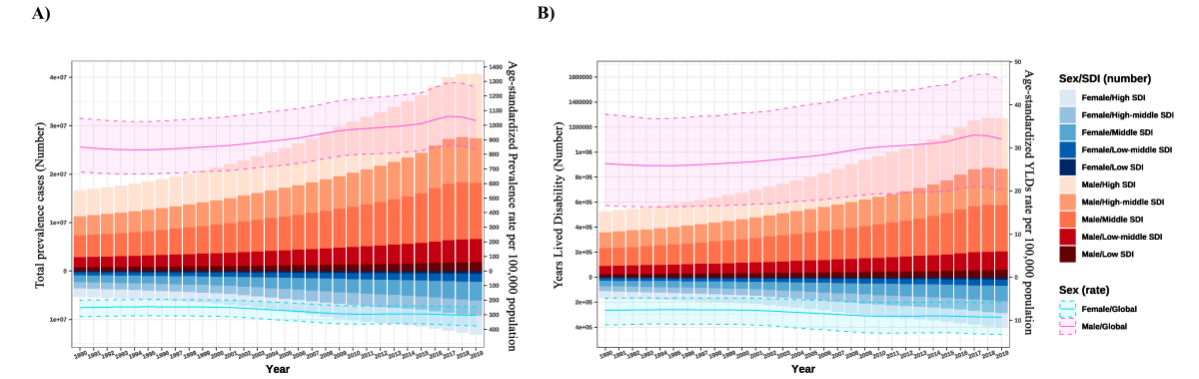
The period effect for gout is shown with all sociodemographic index (SDI) quantiles on (A) total prevalence cases (number) and age-standardized prevalence rate per 100,000 population and (B) years lived with disability (YLD; number) and standardized YLD rate per 100,000 population. The global tendency under the age effect on both sexes is shown as line charts.

**Figure 3:**
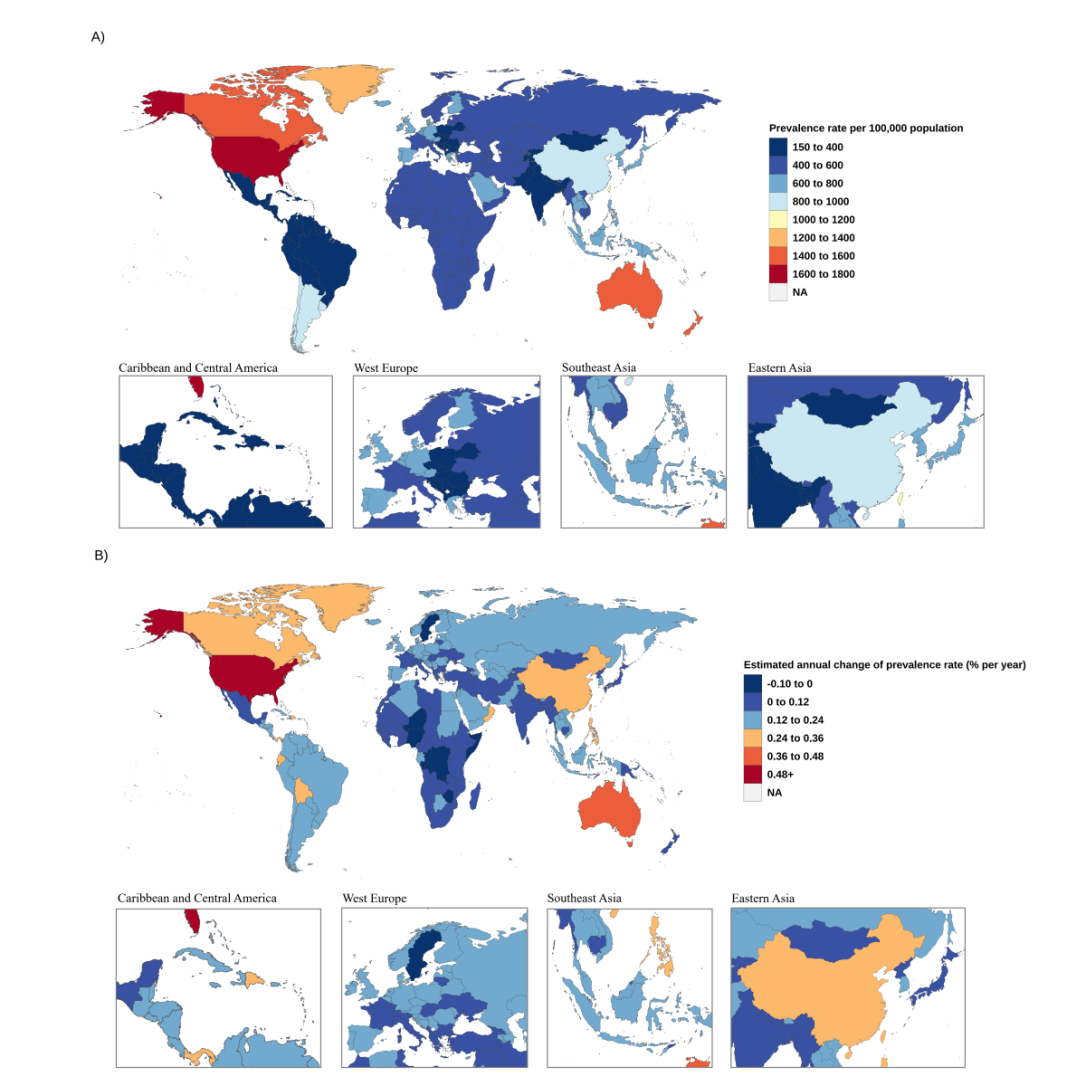
(A) The all-age prevalence for gout in 2019 in 204 countries and territories. (B) Net drift of gout prevalence from 1990 to 2019 in 204 countries and territories.

The gout prevalence trends are similar to the incidence trends. The global all-age prevalence rate increased from 412.42 (95% UI 327.46-511.67) to 696.25 (95% UI 857.42-6634.23) per 10,000 population, and the age-standardized prevalence rate increased from 532.99 (95% UI 425.35-657.86) to 652.24 (95% UI 528.56-798.60) per 10,000 population (Figure 2; Table 1). The high-SDI quantiles have the highest increase in prevalence from 711.83 (95% UI 565.01-887.95) to 1041.01 (95% UI 862.79-1244.91) per 10,000 population, an increase of 47.95%, followed by the high-middle–SDI quantiles, where the age-standardized prevalence increased from 492.64 (95% UI 393.93-611.45) to 624.81 (95% UI 495.85-778.15) per 10,000 population. The prevalence depends on the regions’ SDI development (Figure 3; Table 1).

### APC Effects on Gout Prevalence

To extend the previous studies, we used the APC model to analyze gout burden [21]. Globally, the prevalence of the age effect increases steadily from the age of 30 years in both sexes. The prevalence rises the most readily in high-SDI quintiles across all age groups compared with other quintiles.

Regarding the period effect, gout prevalence witnessed a growth tendency across most SDI quintiles over the study period, with only low-SDI countries remaining nearly constant over the past 2 decades. High-SDI, high-middle–SDI, and middle-SDI countries had the most notable increase in period prevalence rates across the period from 1990 to 2019. By contrast, low-SDI and low-middle–SDI countries stayed relatively stable in terms of prevalence rates. Surprisingly, in terms of the period effect, the gout prevalence rate in female individuals was higher than that in male individuals across the period from 2003 to 2015 in low-middle–SDI, middle-SDI, and high-middle–SDI countries. Still, the data showed that the gout prevalence rate in male individuals was higher than that in female individuals (Figure 1).

**Figure 4:**
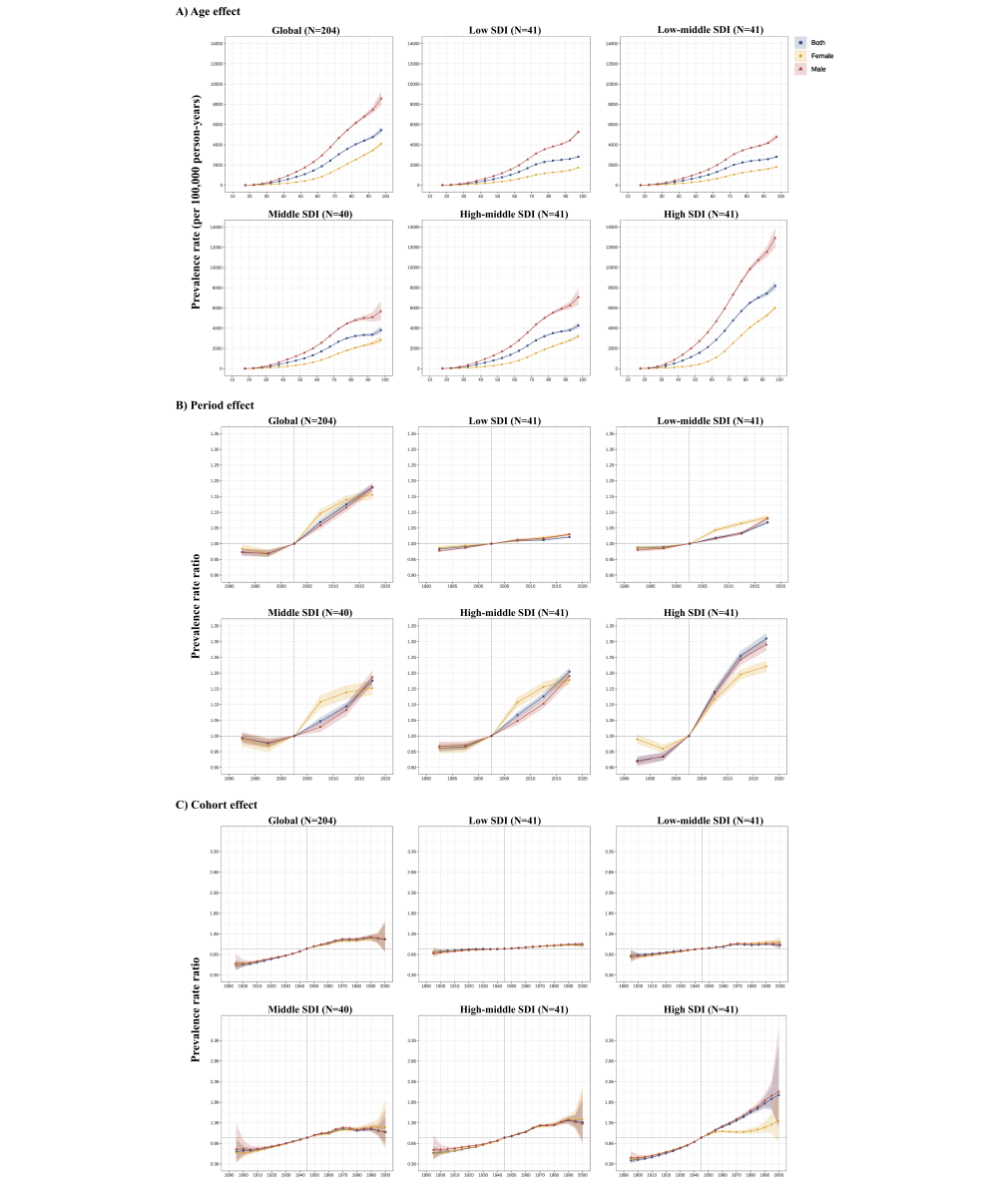
Age-period-cohort effects on gout prevalence by sociodemographic index (SDI) quintiles. (A) Age effects are shown by the fitted longitudinal age curves of prevalence (per 100,000 person-years) adjusted for period deviations. (B) Period effects are shown by the relative risk of prevalence (prevalence rate ratio) and computed as the ratio of age-specific rates from 1990 to 1994 (the referent period) to 2015-2019. (C) Cohort effects are shown by the relative risk of prevalence and computed as the ratio of age-specific rates from the 1925 cohort to the 2015 cohort, with the referent cohort set at 1960. The dots and shaded areas denote prevalence rates and prevalence rate ratios, respectively, and their corresponding 95% CIs.

The morbidity is increased and the younger age population is readily exposed to the risk factor. The growth of cohort effects was remarkable in high-SDI countries. High-SDI countries had progressively increasing morbidity rates among those born after 1900. The sex ratio has shifted in middle-SDI and high-middle–SDI countries, where female individuals have a higher morbidity rate than male individuals in terms of the cohort effect (Figure 4).

### APC Effects in Typical Countries or Regions

**Figure 5:**
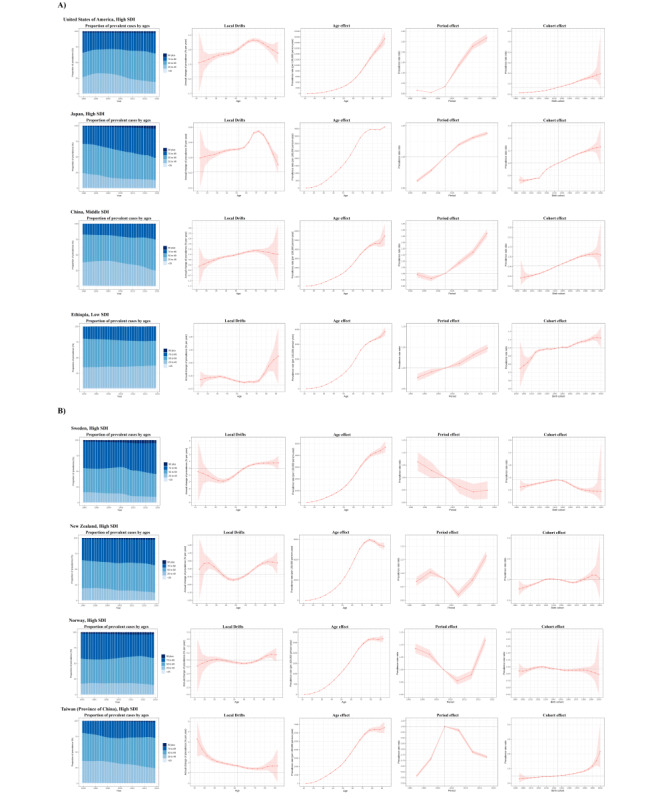
Favorable (A) and unfavorable (B) age-period-cohort effects on exemplar countries across sociodemographic index (SDI) quintiles. Age distribution of deaths shows the relative proportion of morbidity from each age group from 1990 to 2019. Local drifts indicate the annual percentage change in prevalence rate (%) across 5-year age groups (from 0-4 to 65-69 years). Age effects are represented by the fitted longitudinal age curves of prevalence (per 100,000 person-years) adjusted for period deviations. Period effects are represented by the relative risk of prevalence (prevalence rate ratio) and computed as the ratio of age-specific rates in each period compared with the referent period from 1990 to 1994. Cohort effects are represented by the relative risk of prevalence (prevalence rate ratio) and computed as the ratio of age-specific rates in each cohort compared with the referent 1960 cohort. The shaded areas indicate the corresponding 95% CIs of each point estimate. See high-resolution image in Multimedia Appendix 1.

Several countries or regions across SDI quintiles displayed significant developments in gout morbidity by APC effects around the world. The United States showed a trend typical of high-SDI countries, where growing gout prevalence was measured across all age groups. In Japan, another example of a high-SDI country, increased morbidity stood out for its notable net drift and demonstrated an emerging transition in the age distribution of morbidity. Moreover, China had a shift in the age distribution of morbidity, with significantly increased risk in those born after 1970, and the gout prevalence increased dramatically after 2003 in this middle-SDI country. Regarding low-SDI countries, Ethiopia showed a similar phenomenon to that in high-SDI countries because the prevalence kept growing over time. People aged <75 years had significant local drift in terms of being diagnosed with gout. People born after 2000 had a significantly higher risk of developing gout (Figure 5A).

Countries displaying relatively unfavorable APC effects on prevalence are shown in Figure 5B. Sweden, a high-SDI country, showed increasing risk throughout the age effect, but the period effect reduced the prevalence. In addition, people born after 2000 in Sweden have a lower risk of developing gout. New Zealand and Norway showed a descending trend in the period effect but rebooted the prevalence rate after 2008. By contrast, Taiwan showed the opposite result, with the prevalence rate consistently decreasing after 2003.

### Trends in Gout Incidence Over Time

**Figure 6:**
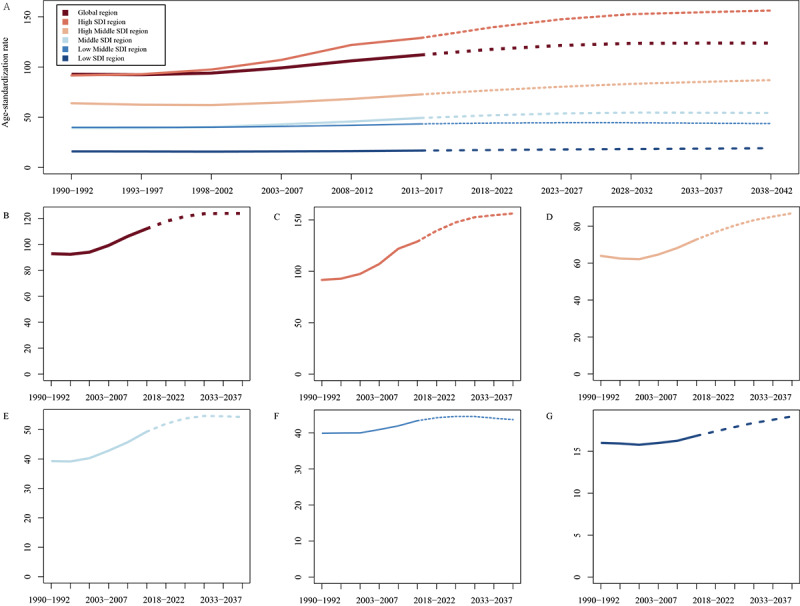
Trends in gout by Nordpred age-period-cohort prediction model in (A) global scale. To minimize the scale bias, the plots were separated as follows: (B) global scale, (C) high–sociodemographic index (SDI) quantiles, (D) high-middle–SDI quantiles, (E) middle-SDI quantiles, (F) low-middle–SDI quantiles, and (G) low-SDI quantiles.

The predicted incidence rates of gout per 100,000 population were calculated using the *Nordpred* APC prediction for different SDI regions (Figure 6). Overall, the predicted incidence rates of gout increased over time in all regions. The global predicted incidence rate increased from 92.79 per 100,000 population in the period from 1990 to 1992 to 123.83 per 100,000 population in the period from 2038 to 2042. The highest predicted incidence rate was observed in the high-SDI quantiles region, with a predicted incidence rate of 156.28 per 100,000 population in the period from 2038 to 2042. The lowest predicted incidence rate was observed in the low-SDI quantiles region, with a predicted incidence rate of 19.16 per 100,000 population in the period from 2038 to 2042. There were also notable differences in the predicted incidence rates among regions; for instance, the predicted incidence rates in the high-middle–SDI quantiles region were consistently lower than those in the high-SDI quantiles region but higher than those in the middle-SDI quantiles and low-middle–SDI quantiles regions. Similarly, the predicted incidence rates in the low-middle–SDI quantiles region were consistently lower than those in the middle-SDI quantiles region but higher than those in the low-SDI quantiles region.

Overall, the number of cases of gout increased over time in all regions. Globally, in 2023, a total of 10,016,336 cases were reported, which is projected to increase to 12,082,807 cases in 2035. The low-SDI quantiles region had the lowest number of cases, with 490,786.4 cases reported in 2018, which is projected to increase to 788,807.5 cases in 2042. There were also notable differences in the number of cases among regions; for instance, the high-SDI quantiles region had the second highest number of cases, with 2,242,423 cases reported in 2018, which is projected to increase to 2,395,031 cases in 2035. The low-middle–SDI quantiles region had a slightly higher number of cases than the middle-SDI quantiles region, but the trend is expected to reverse by 2035, with the middle-SDI quantiles region projected to have more cases (Multimedia Appendix 2).

**Figure 7:**
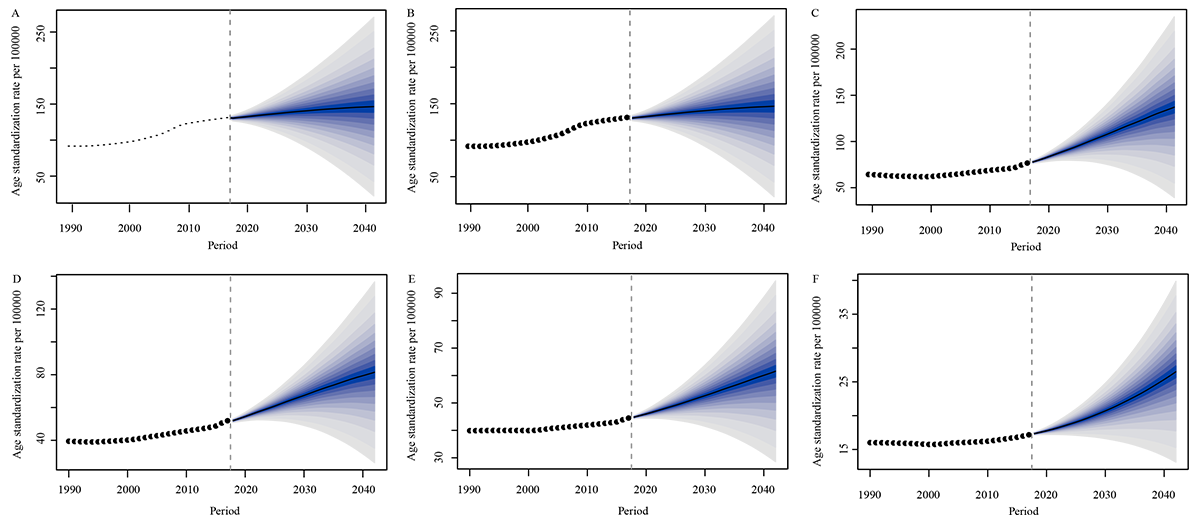
Trends in gout by Bayesian age-period-cohort prediction model in (A) global scale, (B) high–sociodemographic index (SDI) quantiles, (C) high-middle–SDI quantiles, (D) middle-SDI quantiles, (E) low-middle–SDI quantiles, and (F) low-SDI quantiles.

The BAPC prediction model was used as a sensitivity analysis to validate the findings from the APC analysis. Consistent with the APC analysis, the BAPC prediction model suggested that there would be an overall increase in the age-standardized incidence of the disease from 1990 to 2042 across all 6 regions. The highest increase in age-standardized incidence is expected to occur in the high-middle–SDI quantiles, with the incidence increasing from 64.30 per 100,000 population to 137.14 per 100,000 population. The high-SDI quantiles are also expected to experience a significant increase in age-standardized incidence, from 91.68 per 100,000 population to 146.51 per 100,000 population. The middle-SDI quantiles are expected to see an increase from 39.44 per 100,000 population to 81.48 per 100,000 population. The low-middle–SDI quantiles are expected to experience an increase from 39.86 per 100,000 population to 61.54 per 100,000 population. The low-SDI quantiles are expected to experience the smallest increase in age-standardized incidence, from 15.99 per 100,000 population to 26.49 per 100,000 population. Overall, the BAPC prediction model suggests that there will be a significant increase in the incidence of the disease across all regions, with the highest increases occurring in regions with higher-SDI quantiles (Figure 7).

## Discussion

### Principal Findings

Gout is among the most common chronic rheumatic and inflammatory arthritis diseases worldwide. Understanding trends in gout prevalence is key for governments to distribute health care resources better [26]. Despite gout having a low mortality rate and high *curable* rates without comorbidities, gout prevalence has steadily increased globally owing to inadequate management by both medical professionals and patients in many countries [27,28]. The net drift in the majority of high-SDI quantiles increased most readily in terms of both period and cohort effects. To our knowledge, this is the first study using the APC model to analyze global trends in gout prevalence, enabling comparisons among different countries and predictions of future incidence. Compared with previous publications [7,21], our study advances the field by providing a more comprehensive and nuanced understanding of gout prevalence trends and contributing to public health knowledge through future burden estimations. To be precise, the analysis of age effects, period effects, and cohort effects allowed us to determine the prevalence trends by period and birth cohort in individual regions, providing an in-depth evaluation of the quality of the health care system with regard to gout management. The model allowed a comparison among countries and the discovery of some countries or regions with effective interventions. By highlighting these responsive quantiles, we hope that inspiration will strike for a novel or potential idea to better manage gout.

This study illustrates the increase in gout prevalence over time from 1990 to 2019. This tendency would not change, according to our APC model prediction. The major challenge was that the highest gout prevalence occurred in the high-SDI quantiles where the quality of medical care is outstanding. This is an indication, perhaps, that advanced interventions and techniques are not sufficient to solve the problem; rather, this points to the need for the entire medical system to work together. With populations increasing worldwide and with an increase in the aging population, the situation regarding gout prevalence is delicately poised. This analysis of the gout burden in different regions, therefore, is important from the point of view of learning how the medical system works in countries that have demonstrated superior management of gout.

A previous study reported the global gout burden during the period from 1990 to 2010 [8]. With a 9-year data gap, it is necessary to perform updated research to learn trends in gout prevalence and identify the challenges our health system may face in the future. In addition, it is essential to evaluate the effectiveness of the gout management strategies used during the period from 2010 to 2019. Compared with the Disease Modelling Meta-Regression model, the APC model could divide data into 3 spatially varying parameters with conditioning. The conditioning order-dependent model was validated on the spatially varying coefficients; therefore, the model was available for the individual country calculations [16]. Consequently, the APC model was more favorable with regard to the Watanabe-Akaike information criterion than the model with independent random effects [29].

Our analysis of gout prevalence estimates suggests that many low- and middle-income countries, especially those with a high SDI, have had an increasing gout prevalence tendency. Still, the low-SDI countries have relatively stable conditions regarding gout prevalence. We also found that people born after 2000 were more likely to develop gout than those born after 1900, suggesting that awareness of health care resources and education for gout are insufficient [7]. It is possible that patients who experience gout flares may be ashamed of admitting their lifestyles, including the excessive intake of purine-rich foods and alcohol, and this may be combined with low health literacy, because of which they do not receive sufficient information on gout [30]. Most patients with gout visit inpatient clinics only for relief of gout flares, and it has been suggested that the weak relationship between the physician and the patient with gout is leading to treatment nonadherence [31,32]. Another possible explanation for the higher likelihood of gout development among individuals born after 2000 compared with those born after 1900 is the improvement in people’s standard of living and dietary habits, which may have led to an earlier onset of gout [33]. This highlights the importance of considering multiple factors when analyzing the trends in gout prevalence and the burden of gout.

Age-standardized prevalence and YLD can provide a clear picture for comparisons among different economic countries or regions. Because of the varying population structure in different SDI countries and regions, we should interpret the results carefully. In lower-SDI countries, citizens tend to have shorter life expectancy and higher birth rates; therefore, the all-age prevalence is relatively lower than the standardized-age prevalence [34,35]. To cope with the actual situation, health care systems may consider investing more resources in *gout flare* services, especially effective treatment. However, the aging population structure is one of the common social issues in higher-SDI countries. Although the standardized-age prevalence is lower than the all-aged prevalence, because older adults have become a prominent part of the population and also because of rising obesity rates, the health burden would keep increasing. Prevention and long-term management are key to coping with the situation [36,37].

Although the standardized-age prevalence of chronic diseases is lower among younger age groups, the increasing prevalence of obesity and the aging population in the United States has contributed to a considerable health burden. As one of the high-SDI countries, the United States has experienced the most substantial incidence and prevalence rates in the world, with an estimated 1 million cases [38]. Although the United States provides one of the best medical services in the world, gout is the most common inflammatory arthritis disease with significant morbidity and mortality [39]. Gout management strategies, including monitoring and treatment options, are far from enough. According to our results, gout prevalence in the United States has yet to plateau. The rates of obesity, hypertension, and chronic kidney disease keep rising because of the aging population and longer life expectancy [40-42]. As a result, the quality of life may not be improved even with advanced medical services.

In contrast to the United States, Japan, a high-SDI country, has a lower prevalence rate, despite a steadily increasing trend. Japanese traditionally favor purine-rich foods, including seafood, miso, soy sauce, and umami broth [43]. A possible reason for Japan having a lower prevalence rate than the United States is the country’s treat-to-target approach to gout [44,45]. The Japanese medical profession has suggested that patients who present with hyperuricemia and those without symptoms should undergo urate-lowering therapy because hyperuricemia is the etiology causing gout flares [46]. This approach is one of the breakthrough ideas for gout prevention. Checking for the presence of urate in the blood during routine examination may be a possible way to control and prevent gout.

China is the world’s second most populous country, with a population of 1.4 billion. The prevalence of gout in China was lower than that of the United States and European countries in 1990, the reasons for which are differences in genetic factors and exposure [7]. In addition, in 1990, China’s public health institutions were undeveloped, and most public health workers had a poor opinion of the whole system [47]. Therefore, it became a challenge to collect national-level data; as a result, detection accuracy was unsatisfactory, and it remained so until the public health system was reformed. Subsequently, because of rapid westernization and urbanization, the Chinese changed their lifestyles; thus, gout prevalence kept rising in this large-population country with higher diagnosis accuracy. However, various degrees of urbanization and levels of quality of hospitals in different regions or provinces could lead to an extensive range of prevalence [48-50]. It has been reported that the obesity rate could reflect gout prevalence indirectly [51]; thus, it may be an index for the public health professional for the surveillance and prediction of gout prevalence. In low and middle tropical countries, the focus is mainly on infectious diseases and parasites, with insufficient epidemiological surveillance. Because of the younger population, the noncommunicable disease burden is underestimated in these regions. After age standardization, the mortality rate in low and middle tropical countries is more severe than that in high-income countries owing to lack of proper management and health education [52]. To better distribute resources, governments should financially support public health institutions to screen for a *curable* chronic disease, which is gout in this case [53].

A few countries such as Sweden and Taiwan showed a descendant trend in gout prevalence in terms of the period effect. However, several studies have reported that gout management in these countries was not satisfactory owing to the low rate of urate-lowering therapy use, long-term management issues, and poor gout management education [54-56]. Kuo et al [56] explained that the phenomenon could be due to statistical bias, including the delay in prevalence reaction time, short follow-up time, and overestimation of incidence rates from previous studies. However, our APC model presented the period effect from 1990 to 2019; therefore, the length of the follow-up period is sufficient. Besides the education level, ethnic differences, income, and occupation, the possible reason for the aforementioned phenomenon is the different levels of diagnostic certainty [26,57]. In addition, these countries have improved health care systems with regard to gout management because the government provides public data for researchers to study the disease. In Sweden, the Skåne Healthcare Register provides nationwide data for rheumatologists to study gout. Therefore, the rheumatologists could analyze the data with different dimensions, including by region and population [54,58-62]. In Taiwan, the researchers focused on the comorbidities caused by gout [63-68]. As mentioned previously, the mortality gap has stayed consistent over 2 decades. Gout complications could also affect the quality of life catastrophically. Research is necessary to understand and analyze the relationship between gout and its complications. It is also essential to explore the correlations between gout and other diseases. These studies are not only for academic use but also for practical use in the future. Although gout prevalence showed a descendant trend in Sweden, the Swedish public health professional is not satisfied regarding the prevalence and incidence of gout in the country [69]. Therefore, awareness should be created of chronic inflammatory arthritis prevalence on a worldwide level, and immediate action should be taken, including gout management education for both patients and healthy populations.

Although gout is a chronic disease, the primary management involves short-term symptomatic treatment [70,71]. Chronic diseases are often established integrated health care systems with multidisciplinary input [72]. It could prevent comorbidity effectively with the advanced policy science [73]. Primary health care for chronic diseases involves different approaches. At outpatient clinics, patients receive comprehensive advice regarding, for example, nutrition intake, individual susceptibility, body weight, and behavior changes. As gout is caused by multiple factors, the individual approach, such as nursing case management, shows significantly positive results [74]. Regarding health care system approaches, a medical record network set up among hospitals can play an important role. A patient’s medical history could track gout flare frequency, which could enable evaluation of the effectiveness of the management strategy. Of note, a patient’s medical history is a record of their lifestyle in the past (eg, alcohol use and body weight). Hence, the establishment of a medical record network may help to provide effective support for, and monitoring of, patients with gout [75]. It has been reported that low medication adherence among patients with gout is due to time constraints, cost, and scarcity of incentives [76]. Technology could help to overcome these barriers; for example, mobile phone apps and personal activity tracker tools could provide a novel approach to helping patients to change health behaviors [77]. Monitoring patients’ health state remotely, improving patient convenience, and protecting patient privacy could enhance patient medication adherence. It may also be meaningful for health care departments to refer to the policy on, and the management of, other common chronic disease (eg, cardiovascular disease, diabetes mellitus, and asthma).

### Limitations

This study includes limitations. First, limited primary data could be obtained for analysis because the data was modeled in a higher setting by the Institute for Health Metrics and Evaluation, an independent global health research center at the University of Washington, which coordinated the GBD Study 2019. Therefore, the results are heavily dependent on the GBD Study 2019 results data. It is essential to ensure the quality of country-level data collection for a health system because the public health professional can evaluate the effectiveness of the local guideline and policy with in-depth studies. Cohort studies and prospective studies are essential for studying health issues in various populations. An epidemiological study could be combined with pharmacology, mental health, etiology, and genetic studies. Second, the APC model was the mathematical approach used. However, it does not fully reflect the realistic situation. Therefore, the results should be interpreted with caution.

### Conclusions

Our study provides important insights into the global burden of gout and highlights the need for effective management and prophylaxis of this condition. The APC model used in our analysis provides a novel approach to understanding the complex trends in gout prevalence and incidence. Gout prevalence in the global population has increased significantly over the past 3 decades, particularly in high-SDI regions. Our study also identified an increasing trend in gout incidence, which is predicted to continue in the coming years. These findings emphasize the urgency of addressing the inadequate strategies for management and prevention of gout in many countries. To cope with the increasing gout prevalence, we recommend the integration of multidisciplinary input and the establishment of medical record networks to improve gout management.

## Appendix

Multimedia Appendix 1 High-resolution image for Figure 5.

Multimedia Appendix 2 Trends in observed (dashed lines) and predicted (solid lines) gout in number of incidence cases in the (A) global scale, (B) high–sociodemographic index (SDI) quantiles, (C) high-middle–SDI quantiles, (D) middle-SDI quantiles, (E) low-middle–SDI quantiles, and (F) low-SDI quantiles. Shading indicates whether the rate remained stable (baseline reference), decreased by 1% per year (optimistic reference, lower limit), or increased by 1% per year (pessimistic reference, upper limit) based on the observed rate in 2019.

## Abbreviations

APC: age-period-cohort
BAPC: Bayesian age-period-cohort
GBD: Global Burden of Disease
ICD-10: International Classification of Diseases, Tenth Revision
ICD-9: International Classification of Diseases, Ninth Revision
INLA: integrated nested Laplace approximations
SDI: sociodemographic index
UI: uncertainty interval
YLD: years lived with disability

## Data Availability

All data were obtained via the Global Burden of Disease 2019 study.
