# Supplementary material for: Global, Regional, and National Prevalence of Gout From 1990 to 2019: Age-Period-Cohort Analysis With Future Burden Prediction
Source: JMIR Public Health Surveill. 2023 Jun 7;9:e45943. doi: 10.2196/45943 (PMC10285625; doi:10.2196/45943)
Supplement: Multimedia Appendix 1 [file publichealth_v9i1e45943_app1.pdf]

A)

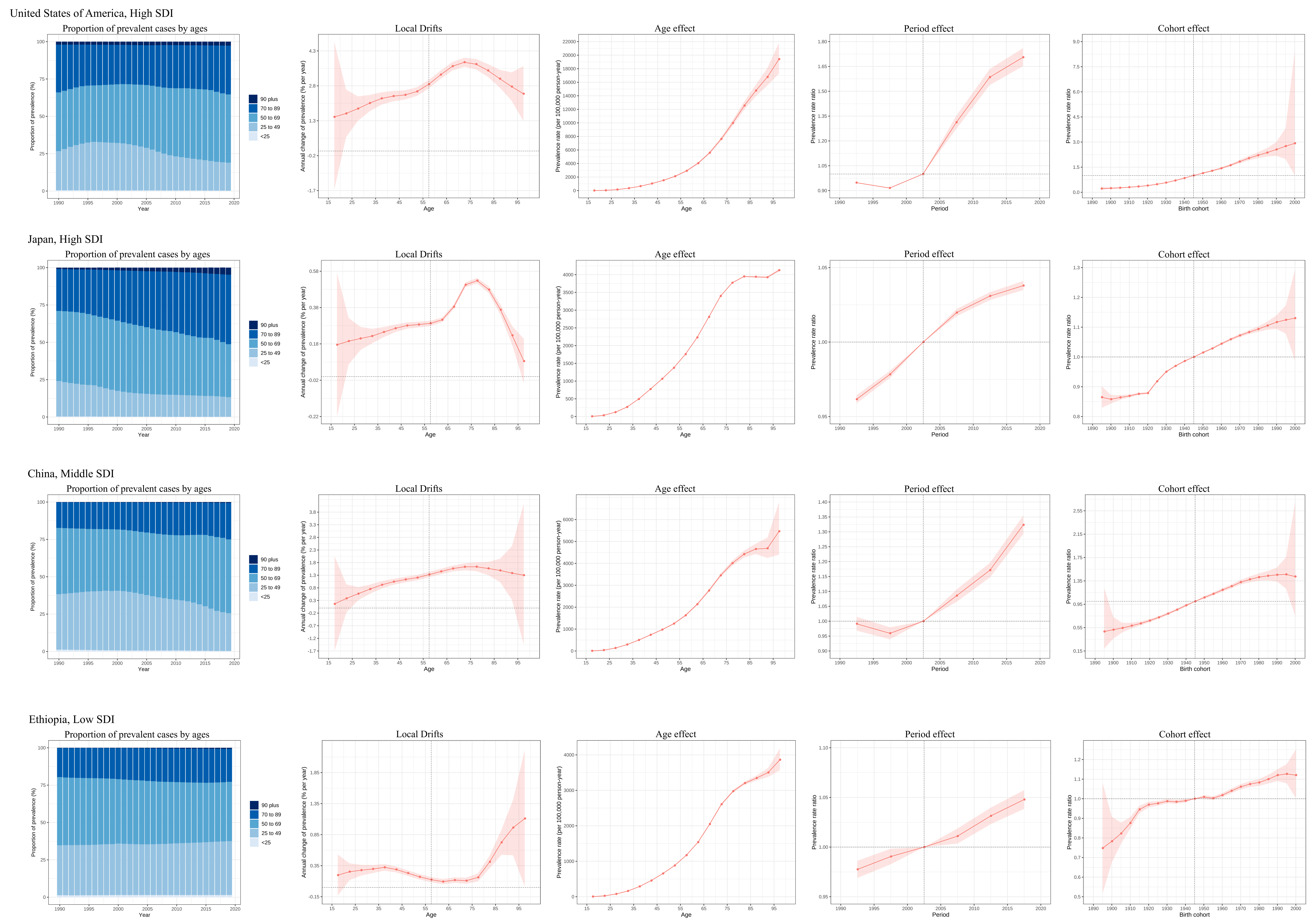
United States of America, High SDI
Japan, High SDI
China, Middle SDI
Ethiopia, Low SDI
Proportion of prevalent cases by ages
Local Drifts
Age effect
Period effect
Cohort effect
Proportion of prevalence (%)
Annual change of prevalence (% per year)
Prevalence rate (per 100,000 person-year)
Prevalence rate ratio
Year
Age
Period
Birth cohort
90 plus
70 to 89
50 to 69
25 to 49
<25

B)

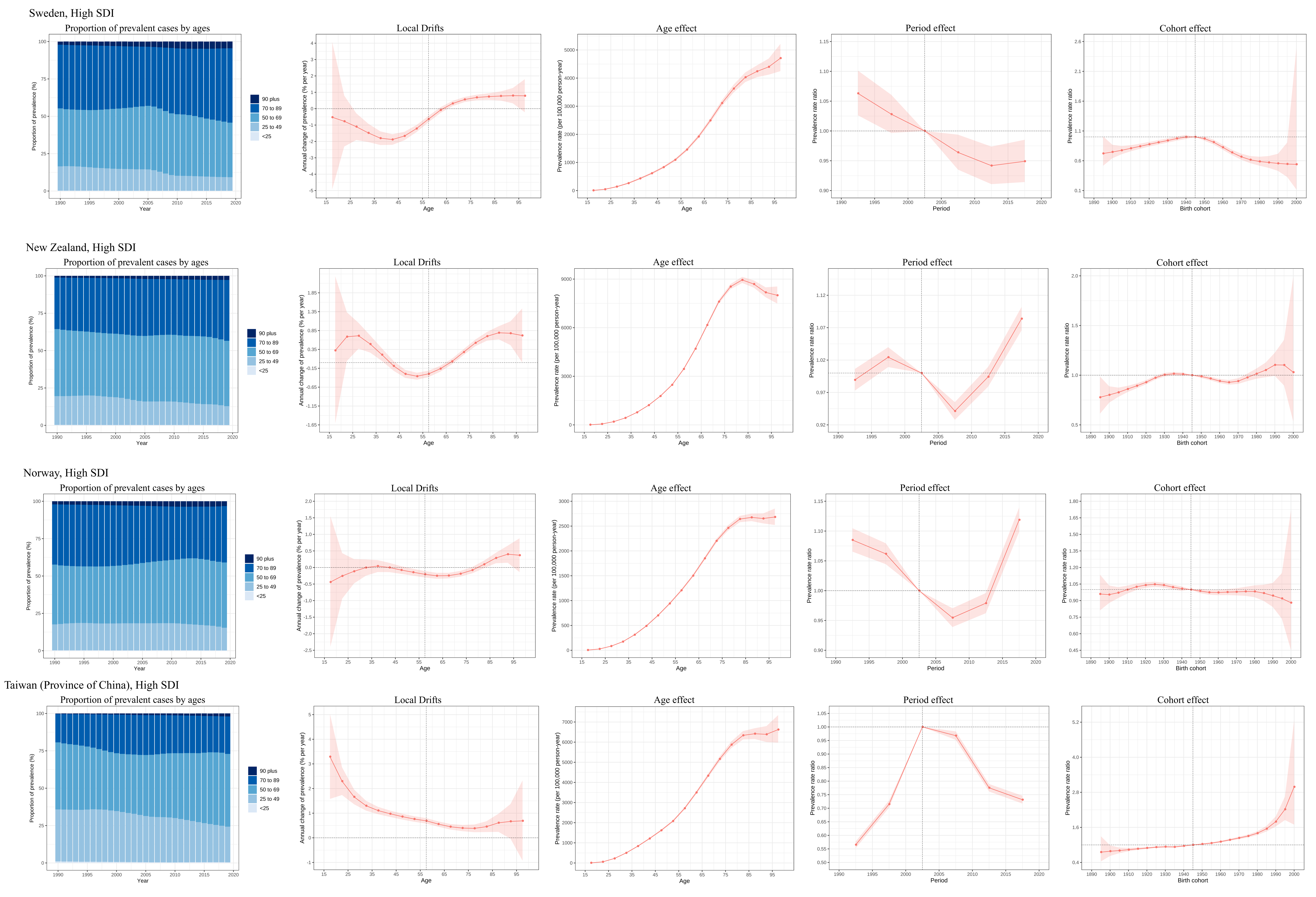
Sweden, High SDI
New Zealand, High SDI
Norway, High SDI
Taiwan (Province of China), High SDI
Proportion of prevalent cases by ages
Local Drifts
Age effect
Period effect
Cohort effect
Proportion of prevalence (%)
Annual change of prevalence (% per year)
Prevalence rate (per 100,000 person-year)
Prevalence rate ratio
Year
Age
Period
Birth cohort
90 plus
70 to 89
50 to 69
25 to 49
<25
