# Supplementary figures and images for: Global, Regional, and National Prevalence of Gout From 1990 to 2019: Age-Period-Cohort Analysis With Future Burden Prediction
Source: JMIR Public Health Surveill. 2023 Jun 7;9:e45943. doi: 10.2196/45943 (PMC10285625; doi:10.2196/45943)

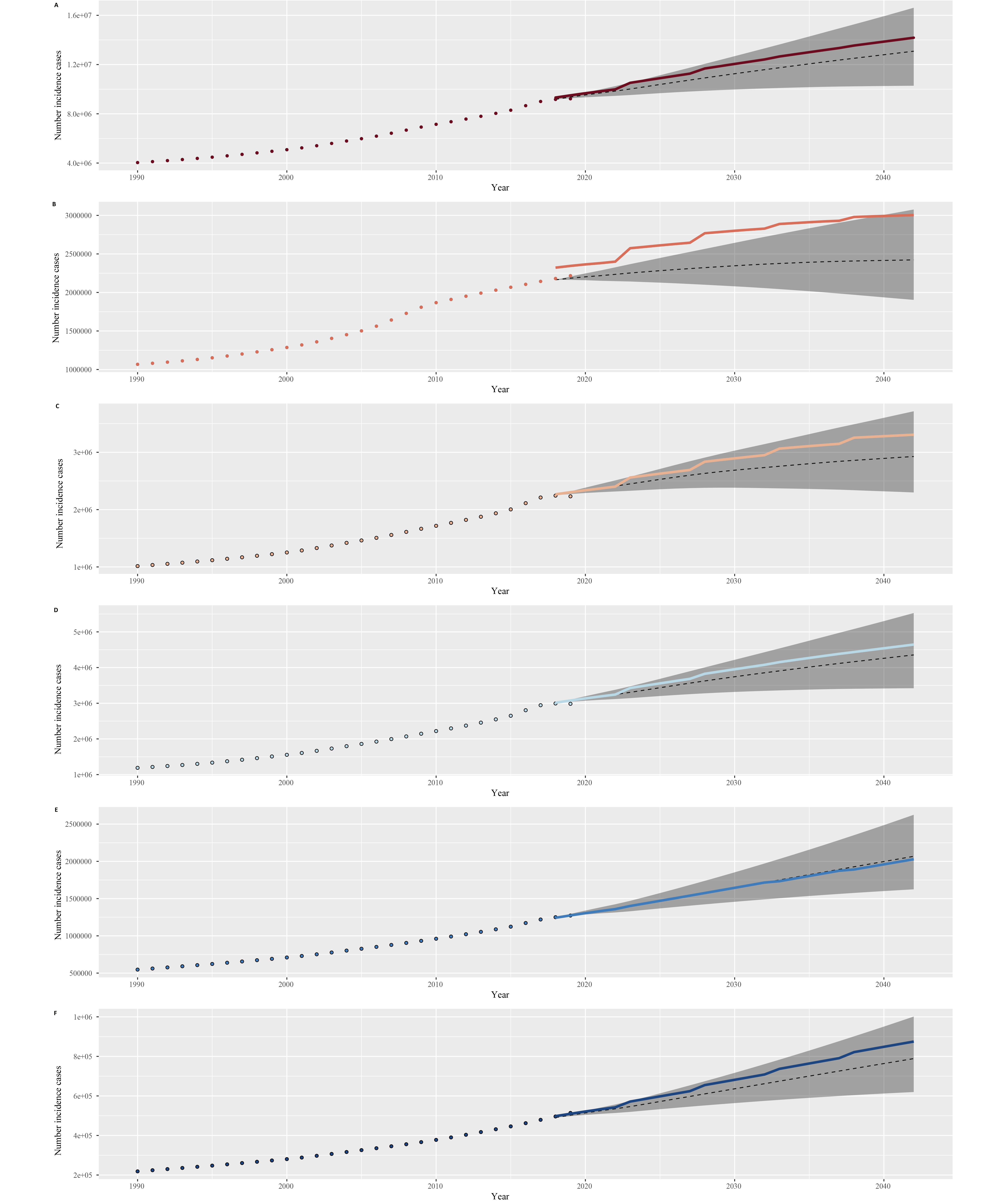

Supplement: Multimedia Appendix 2 [file publichealth_v9i1e45943_app2.png]
